# A Bioluminescent Biosensor for Quantifying the Interaction of SARS-CoV-2 and Its Receptor ACE2 in Cells and In Vitro

**DOI:** 10.3390/v13061055

**Published:** 2021-06-02

**Authors:** Xiaolong Yang, Lidong Liu, Yawei Hao, Eva So, Sahar Sarmasti Emami, Derek Zhang, Yanping Gong, Prameet M. Sheth, Yutian Wang

**Affiliations:** 1 Department of Pathology and Molecular Medicine, Queen’s University, Kingston, ON K7L 3N6, Canada; yh5@queensu.ca (Y.H.); sahasarmasti1990@gmail.com (S.S.E.); 16dyz@queesnu.ca (D.Z.); yanping.gong@kingstonhsc.ca (Y.G.); ps70@queensu.ca (P.M.S.); 2 DM Center for Brain Health and Department of Neurology, Faculty of Medicine, University of British Columbia, Vancouver, BC V6T 1Z3, Canada; lidong@mail.ubc.ca (L.L.); evaso@mail.ubc.ca (E.S.); ytwang@brain.ubc.ca (Y.W.); 3 Gastrointestinal Disease Research Unit (GIDRU), Faculty of Health Science, Queen’s University, Kingston, ON K7L 3N6, Canada

**Keywords:** COVID-19, SARS-CoV-2, ACE2, RBD, NanoBiT, bioluminescent biosensor, therapeutic drugs

## Abstract

The severe acute respiratory syndrome coronavirus-2 (SARS-CoV-2) is currently spreading and mutating with increasing speed worldwide. Therefore, there is an urgent need for a simple, sensitive, and high-throughput (HTP) assay to quantify virus–host interactions in order to quickly evaluate the infectious ability of mutant viruses and to develop or validate virus-inhibiting drugs. Here, we developed an ultrasensitive bioluminescent biosensor to evaluate virus–cell interactions by quantifying the interaction between the SARS-CoV-2 receptor binding domain (RBD) and its cellular receptor angiotensin-converting enzyme 2 (ACE2) both in living cells and in vitro. We have successfully used this novel biosensor to analyze SARS-CoV-2 RBD mutants and evaluated candidate small molecules (SMs), antibodies, and peptides that may block RBD:ACE2 interaction. This simple, rapid, and HTP biosensor tool will significantly expedite the detection of viral mutants and the anti-COVID-19 drug discovery process.

## 1. Introduction

The recent outbreak of coronavirus SARS-CoV-2 caused the human respiratory disease called COVID-19 in China and worldwide. At the time of writing, over 149 million cases and 3.1 million deaths have been attributed to the virus. The key in the fight against this virus is the development of fast, accurate, sensitive diagnostic tools, and potent therapeutic drugs. Most recently, multiple tools, which include the fast detection of viral mRNAs and anti-viral antibodies (IgM and IgG) in COVID19+ patients, have been developed [1]. However, drugs effectively eradicating the virus are still lacking. Currently, the most promising approach to stop the virus is to develop vaccines against this virus.

Although various vaccines have been developed and shown to be effective to prevent COVID-19 infection, the SARS-Cov-2 virus continues to mutate to escape vaccines. Therefore, it is also possible that it will become endemic and circulate in the population like the seasonal influenza virus (flu). Thus, for the long-term fight against COVID-19, other long-lasting therapeutic drugs targeting this virus are also required. 

In addition to vaccines, two major approaches have been proposed or reported to target SARS-CoV-2 [2]: (1) The use of SMs, antibodies or peptides to inactivate proteins involved in viral entrance (e.g., viral Spike (S) protein (anti-S antibodies)), processing (e.g., cleavage by proteases M^pro^ and 3CL^pro^ (Lopinavir-ritonavir), and endocytosis (e.g., hydroxychloroquine)), and replication (e.g., RNA polymerase (remdesivir)). (2) The use of convalescent plasma/sera containing anti-SARS-CoV-2 antibodies from recovered patients.

However, up until now, there have been no developed drugs used in clinical treatment that can effectively treat COVID-19 to result in reduced mortality. Since clinical trials usually take years to accomplish, there is an urgent need for tools that can quickly test the effectiveness of candidate anti-virus drugs to expedite the discovery processes. In addition, although many SARS-CoV-2 mutants have been detected in new COVID-19 patients globally [3], there is no simple, fast, and accurate way to test whether these mutants will affect their entrance into cells via changed interactions with ACE2 on the cell membrane.

Research reported that SARS-CoV-2 enters human epithelial cells by using the RBD of its S protein to attach to a receptor called the ACE2 protein on the cell surface [4]. Therefore, blocking RBD:ACE2 protein–protein interactions (PPIs) will be an excellent strategy to curb virus entrance/invasion into cells and their subsequent infection [5]. The gold standard to test the effect of RBD/ACE2 mutation, drugs, or antibodies on their interactions is to use viral neutralization assays using either live virus [6] or viral vectors pseudotyped with the S protein [7].

However, these in vivo virus-based assays require a level III biosafety facility and are time-consuming to run. Although some in vitro protein-based assays, such as surface plasmon resonance (SPR) [8] and ELISA [9,10,11], have also been developed in analyzing RBD:ACE2 interactions, they are less sensitive (color detection) and technically challenging (requiring the purification of proteins from mammalian cells and multiple steps or special equipment to perform), which makes them difficult to operationalize in most research and clinical labs. Therefore, a simple, sensitive, and fast assay that can be used to perform HTP screening or validation for drugs blocking RBD-ACE2 PPIs for anti-COVID-19 therapy is required.

In 2016, an ultrasensitive split luciferase complementary assay (SLCA) called NanoLuc Binary Technology (NanoBiT) was developed to monitor PPIs [12]. This assay is based on the observation that the NanoLuc luciferase enzyme can be separated into two fragments, an 18-kDa Large BiT (LgBiT or Lg) and 11-amino acid (aa) small BiT (SmBiT or Sm) with each fragment fused to a target protein of interest. The PPIs of these two proteins can lead to the reconstitution of active NanoLuc luciferase from the LgBiT and SmBiT and emit light in the presence of its substrate furimazine.

By using this technology, we successfully constructed a NanoBiT YAP:TEAD biosensor and successfully used it to screen and identify SMs disrupting YAP-TEAD PPIs in cancer therapy [13]. In this study, we used this simple and powerful technology and developed the first NanoBiT biosensor to quantify SARS-CoV-2 RBD:ACE2 PPI both in vitro and in vivo. We also validated this biosensor to test the effects of SMs, antibodies, and peptides on RBD:ACE2 PPIs.

## 2. Materials and Methods

Chemicals were purchased from Sigma-Aldrich (Oakville, ON, Canada) or Bioshop (Burlington, ON, Canada) unless otherwise stated.

### 2.1. Biosensor Plasmid Construction and In Vitro Mutagenesis

**Figure 1:**
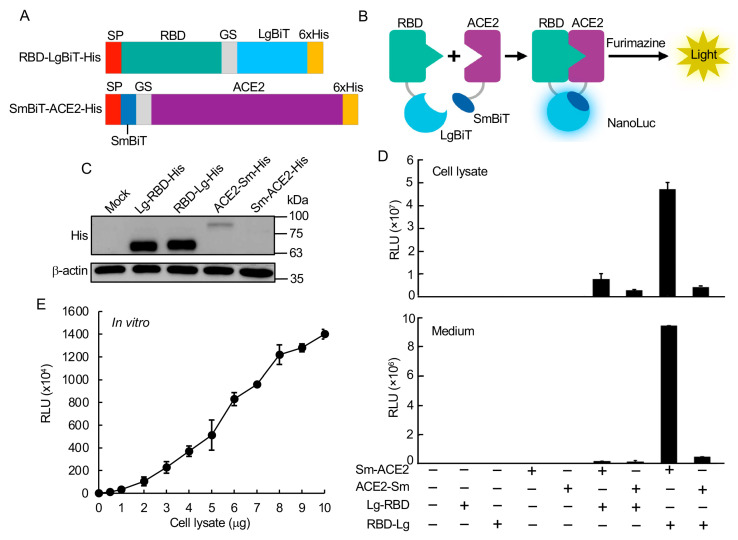
Construction and validation of RBD:ACE2 NanoBiT biosensor. (**A**) Schematic of SRAE2-BS constructs. SP, signal peptide. (**B**) Demonstration of SRAE2-BS working mechanism. (**C**) Western blot analysis of protein expression of SRAE2 components. Plasmids were transfected into HEK293T cells in 12-well plate. 10 µg of protein lysates were subjected to SDS-PAGE, followed by western blot analysis using anti-His first antibody. b-actin was used as internal protein loading control. Molecular weight in kilodalton (kDa) is shown on the right of the blot. (**D**) Luciferase analysis of different combinations of biosensor components. Plasmids were transfected into HEK293T cells in 96-well plate, followed by luciferase analysis using cell lysates from proteins extracted from transfected cells (upper panel) or secreted protein from cell culture medium (lower panel). RLU, relative luciferase unit. (**E**) Luciferase analysis of SRAE2-BS activity in vitro. Equal amounts (0–10 µg) of Sm-ACE2-His and RBD-Lg-His protein lysates were mixed and incubated together for 30 min, followed by luciferase analysis.

To construct the SARS-CoV-2 RBD:ACE2 biosensor (SRAE2-BS), RBD (aa. 319–685) or ACE2 ectodomain (aa. 18–615) was first amplified using PCR from codon optimized SARS-Cov-2 (2018-nCoV) Spike S1 ORF (SinoBiologicals Inc., Beijing, China) or human ACE2 cDNAs (SinoBiologicals Inc.; accession number NM_021804.1), respectively, using PrimStar DNA polymerase (Takara Bio, Mountain View, CA, USA). LgBiT, SmBiT, GS linker (GSSGGGGSGGGGSSG), or a 6 × His tag (Figure 1A) was added to either the N- or C-terminal PCR products of the RBD or ACE2 by including their sequences in primers or by overlapping PCR (see Table S1 for the primer sequences). 

The amplified PCR products were digested with EcoRV/NicoI and subcloned into pFUSE_hIgG1_Fc2 mammalian expression vector (Invivogen, San Diego, CA, USA), which contains a N-terminal IL2 secretion signal peptides (SP). PCR was used to construct SRAE2-BS plasmids with ACE2 or RBD deletions, whereas overlapping PCR was used to make in vitro mutagenesis. The primer sequences are described in Table S2.

### 2.2. Cell Culture

HEK293T (human embryonic kidney) cells were cultured in Dulbecco’s modified Eagle’s medium (DMEM; D6429; Sigma-Aldrich, Oakville, Canada) containing 10% FBE, and 1% Penicillin/Streptomycin (Invitrogen) at 37 °C with 5% CO_2_.

### 2.3. DNA Transfection and Western Blot Analysis

Approximately 1 µg/well of SRAE2-BS plasmid was transfected into HEK293T cells on a 6-well plate using PolyJet transfection reagent (SignaGen, Frederick, MD, USA). Two days after transfection, protein was extracted using RIPA lysis buffer (50 mM Tris.HCl, 150 mM NaCl, 0.02% sodium azide, 1% NP-40, 0.1% SDS, and 0.5% sodium deoxycholate). A total of 10–20 µg of extracted protein lysates was separated using 10% SDS-polyacrylamide gel electrophoresis (PAGE)(Bio-Rad) and transferred to nitrocellulose membranes. The blot was first probed with anti-6 × His mouse monoclonal antibodies (ab18184; Abcam, Cambridge, MA, USA), followed by incubation with goat anti-mouse IgG secondary antibody (Jackson ImmunoResearch). The signal was detected using an ECL chemiluminescence substrate and visualized using an Amersham Imager 600UV.

### 2.4. Peptide Design and Synthesis

The interrupting peptides were designed based on the analysis of the binding sequences between the SARS-CoV2 spike glycoprotein and ACE2, particularly, the amino acid residues between 446 and 505 on RBD and residues 19–393 on ACE2, which was believed to form hydrogen bonds with ACE2 residues 19–393 at the SARA-CoV-2 RBD/ACE-2 interface [8,14,15]. The final sequences of the modified peptides were optimized by using the peptide–protein docking software, HPEPDOCK [16] and HADDOCK [17]. The peptides with the top docking energy scores were selected for the biosensor binding assay.

Peptides were synthesized on a Liberty Blue microwave-aided peptide synthesizer (CEM Corporation, Matthews, NC, USA) using solid phase methodology and Fmoc-protecting group strategy on a Rink Amide MBHA resin (Gyros Protein Technologies, Warren, NJ, USA). In each peptide-build-up cycle, 20% piperidine in DMF (N,N-dimethylmethanamide) was used for N-terminal Fmoc-deprotection and N,N′-diisopropylcarbodiimide (DIC)/Oxyma were used as coupling reagents. Synthesized peptides were cleaved from resin in a TFA cleavage cocktail containing trifluoroacetic acid, phenol, deionized water, thioanisole, and 1,2-ethanedithiol (82.5:5:5:5:2.5%, *v*/*v*) for 2 h at room temperature and precipitated with ice-cooled methyl tert-butyl ether. Crude peptides were further purified on an Agilent 1200Preparative HPLC coupled with 6100 Mass Spectrometry system (Agilent Technologies, Santa Clara, CA, USA) using a reverse phase C-18 column (ACE C18-300, 250 × 21.2 mm, 10 µm) with water/acetonitrile gradient elution. The final purity of all peptides is >95%.

### 2.5. NanoLuc Luciferase (NanoBiT) Assay

For the analysis of biosensor activity in living cells in vivo, 2 × 10^4^ HEK293T cells were seeded in triplicate in a 96-well plate 24 h before transfection. We transfected 100 ng of Lg-RBD, RBD-Lg, Sm-ACE2, or ACE2-Sm plasmids alone or together into HEK293T cells using PolyJet transfection reagent (SignaGen, Frederick, MD, USA). At 24 h after transfection, the medium was transferred to new wells. The attached cells were lysed in 20 µL of 1 × passive lysis buffer (1 × PLB, Promega) at room temperature (RT) for 15 min. We subjected 20 µL of cell lysate or medium to a NanoLuc luciferase assay using Nano-Glo Live Cell Reagent containing 1/50 diluted furimazine substrate (Promega, Madison, WI, USA). Relative Luminescence Unit (RLU) was measured using GloMax Navigator Microplate Luminometer (Promega).

For analysis of biosensor activity in vitro, 500 ng/well of wild-type (WT) or mutant (deletions or point mutations) Sm-ACE2 or RBD-Lg were transfected into 12-well plates using PolyJet transfection reagent (SignaGen). Two days after transfection, the cells were lysed in 1 × PLB at RT for 15 min. Protein concentrations were quantified using a RC DC Protein Assay kit (Bio-Rad, Mississauga, Canada).

Protein lysates were diluted into 1 µg/µL and kept at −80 °C. For in vitro biosensor analysis, equal amounts of WT or mutant RBD-Lg and Sm-ACE2 were mixed together in 10 µL and incubated at RT for 30 min. We added 10 µL of 1/50 diluted furimazine substrate, followed by measurement of the RLUs using GloMax Microplate luminometer. All experiments were repeated at least two to three times. The means and standard deviations (S.D.) of the RLUs of triplicate samples are shown.

### 2.6. Validation of SRAE2-BS Using SMs, Antibodies, and Peptides

Approximately 6 µg of Sm-ACE2 or RBD-Lg plasmids were transfected onto a 100 mm plate using PolyJet. Two days after transfection, protein lysates were extracted, quantified, diluted, and stored as described above. For examining the effect of candidate drugs on biosensor activity, triplicates of increasing amounts (0–1000 µM) of SMs (Baicalin, Hesperidin, Sculellarin, Theaflavin, Heparin from TargetMol) or freshly prepared synthesized peptides (ACE2-P1–2) or 1–50 µg/mL of IgG or VHH72 anti-SARS-CoV-2 Spike RBD LIamabody monoclonal antibody (R&D#LMAB10541) were preincubated with 1 µg of Sm-ACE2 protein lysate in 10 µL in 96-well plates on a rocker at RT for 30 min.

We added 1 µg/10 µL of Sm-ACE2 protein lysates, and the mixture was incubated on a rocker at RT for another 30 min. For peptides targeting ACE2 (RBD-P1~3), increasing concentrations (1–100 µM) of peptides were preincubated with Sm-ACE2 protein lysate for 30 min, followed by incubation with RBD-Lg protein lysates. We added 10 µL of 1/50 diluted furimazine substrate into each well. The mixtures were incubated on a rocker for 2 min followed by measurement of the RLUs using a GloMax Microplate Luminometer (Promega). All experiments were repeated at least twice. The mean and standard error (S.E.) of the triplicate samples were calculated.

### 2.7. Statistical Analysis

Student’s *t*-test (two-tailed) was used for statistical analysis. A value of *p* ≤ 0.05 was accepted as statistically significant.

## 3. Results and Discussion

To build the NanoBiT bioluminescent biosensor monitoring the RBD:ACE2 PPI, we first constructed biosensor plasmids by fusing LgBiT or SmBiT with RBD or ACE2, respectively, at their N- or C-termini. The interaction of RBD and ACE2 will complement LgBiT and SmBiT to form NanoLuc luciferase, which will emit light in the presence of its substrate furimazine (Figure 1B). Western blot analysis of each construct showed that, while similarly high levels of Lg-RBD-His and RBD-Lg-His were expressed in cells, relatively low levels of Sm-ACE2-His and ACE2-Sm were expressed (Figure 1C). 

The luciferase analysis of biosensor activity indicated that transfection of each component of the biosensor alone had little luciferase activity, whereas the combination of Sm-ACE2 and RBD-Lg obtained the highest activity in both the protein lysate extracted from cells transfected with plasmids and the collected cell culture medium (secreted biosensor) (Figure 1D). To analyze the biosensor activity in vitro, we combined increasing amounts (0–10 µg) of Sm-ACE2 protein lysates with equal amounts of RBD-Lg lysates in vitro and incubated at RT for 30 min, followed by luciferase analysis. 

Significantly, the combination of only 1 µg of protein lysate of Sm-ACE2 and RBD-Lg obtained over 1 × 10^5^ RLU. The signal intensity linearly increased with increasing amounts of added cell lysates (Figure 1E). These data strongly suggest that we were able to quantify RBD-ACE2 PPI both in vivo in cells and in vitro using protein lysates extracted from plasmid-transfected cells. This newly developed bioluminescent biosensor tool is simple (only a luciferase assay in vitro), sensitive (~0.2–1 µg crude cell lysate per assay; no protein purification is required), fast (30 min in vitro), accurate (quantified by light emission unit), HTP (96-well or 394-well plate), and cheap (<1$ per assay).

**Figure 2:**
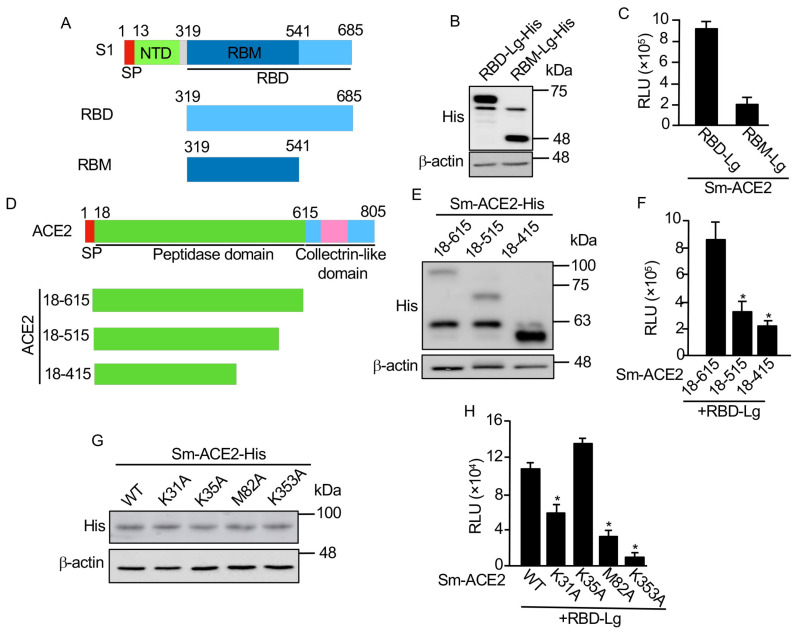
Mapping minimum region and critical residues for RBD:ACE2 PPI. (**A**) Schematic of RBD deletions. RBD, receptor binding domain; SP, signal peptide; NTD, N-terminal domain; RBM, receptor binding motif. (**B**,**E**,**G**) Western blot analysis of protein expression. (**C**,**F**,**H**) In vitro luciferase assay. Equal amounts (1 µg) of each biosensor component were incubated together for 30 min, followed by luciferase assay; (**D**) Schematic of ACE2 deletions. Experimental procedures and labels are as described in Figure 1. *, *p* < 0.05.

We are going to use this biosensor to test the functional domains of RBD interacting with ACE2. Previous studies indicated that the receptor binding motif (RBM) in the RBD (Figure 2A) was more important regarding its interaction with ACE2 [18]. We made a RBM-Lg construct and compared its activity with that of RBD in vitro. Although RBD-Lg and RBM-Lg were expressed at similar levels in cells (Figure 2B), compared to RBD-Lg/Sm-ACE2, the biosensor activity of RBM-Lg/Sm-ACE2 in vitro was only 20% of that of RBD-Lg/Sm-ACE2, suggesting that other sequences downstream of RBM (aa. 541–685) are also important for its interaction with ACE2.

A previous structural study showed that the peptidase domain or ectodomain (aa. 18–615) of ACE2 (Figure 2D) is important for its interaction with RBD [8]. However, the minimum region required for the interaction of ACE2 with RBD has not been mapped. To map the minimum region of ACE2 critical for its interaction with RBD, we further truncated the protease domain (Figure 2D). Our biosensor analysis showed that, although similar protein levels were detected for Sm-ACE2-18–615 and Sm-ACE2-18–515 (Figure 2E), the biosensor activity of Sm-ACE2-18-515/RBD-Lg was significantly lower than that of Sm-ACE2-18-615/RBD-Lg (Figure 2F).

Further ACE2 deletion (Sm-ACE2-18–415) stabilized the ACE2 protein (Figure 2E) but reduced biosensor activity (Figure 2F), suggesting that aa. 18–615 of ACE2 has the full-length RBD-binding domain. Based on the structure of the ACE2-RBD complex [14], we also mutated four residues on ACE2 that are important for the binding of ACE2 to RBD. Although similar levels of proteins were expressed for both WT and mutant ACE2 (Figure 2G), the in vitro luciferase assay showed that mutation of ACE2 K31 or M82 into alanine (A) significantly reduced biosensor activity (Figure 2H). Furthermore, the mutation of ACE2 K353 into alanine almost abolished biosensor activity (Figure 2H). These findings not only confirmed the specificity of our SRAE2-BS but also identified K353A as a critical residue for RBD:ACE2 PPI.

**Figure 3:**
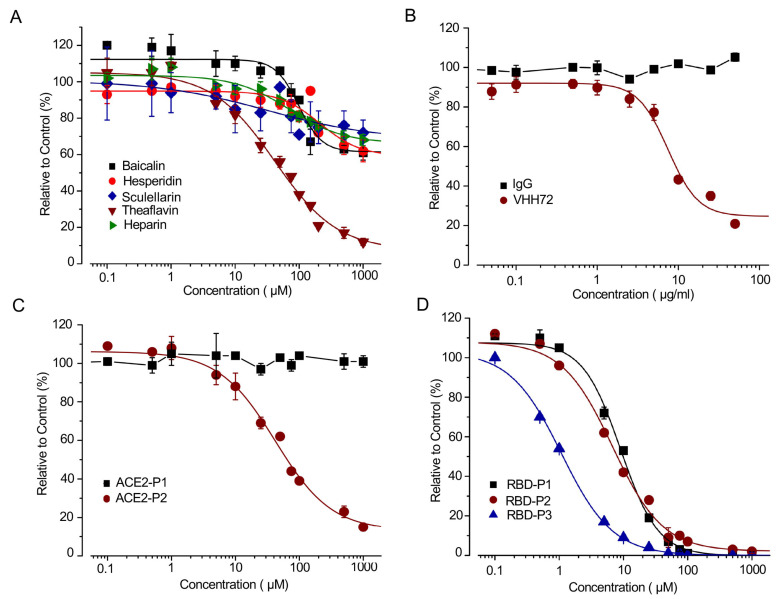
Dose-dependent effect of SMs, antibody and peptides on SRAE2-BS activity. (**A**–**C**) Increasing concentrations of SMs (**A**), IgG (control) or VHH72 anti-RBD antibody (**B**), or ACE2-P1-P2 peptides (**C**) were preincubated with RBD-Lg-His, and subsequently incubated with Sm-ACE2-His for 30 min, followed by measurement of RLU. (**D**) Increasing concentration of RBD-P1-P3 peptides were preincubated with Sm-ACE2-His, and subsequently incubated with RBD-Lg-His, followed by measure of RLU. The mean +/− standard error (S.E.) of relative values (relative to control) of triplicate samples were calculated in comparison to samples without treatment (control).

Currently, no SMs disrupting RBD:ACE2 PPI have been used for COVID-19 clinical treatments. Most studies predict inhibiting RBD:ACE2 PPI using computer modeling [5,19,20]. We used our SRAE2-BS to test several candidate SMs (i.e., Baicalin, Hesperidin, Sculellarin, Theaflavin, and Heparin) predicted by computer modeling to be able to disrupt RBD-ACE2 PPI [19,20,21]. Surprisingly, out of the five SMs tested, only Theaflavin significantly suppressed SRAE2-BS activity with an IC50 of 41.7 µM (Figure 3A). This result suggests that a functional analysis, such as our biosensor assay, is required to finally validate the true SMs disrupting RBD-ACE2 PPI.

**Figure 4:**
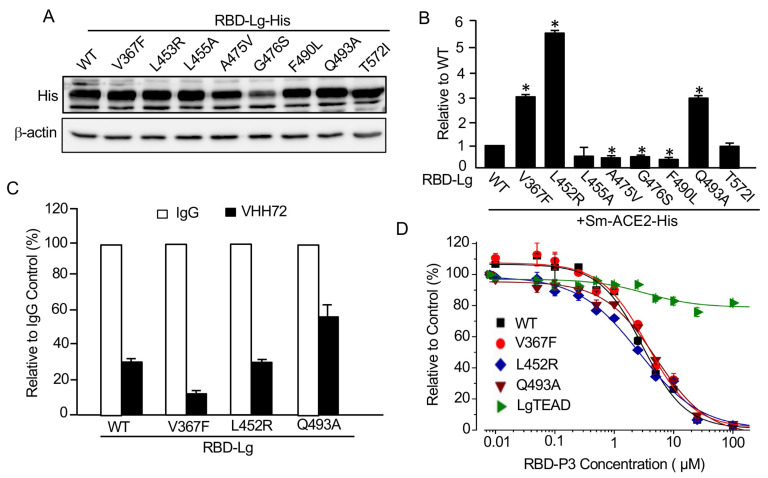
Mutations of SARS-CoV-2 RBD on its interaction with ACE2 and response to antibody and peptides. (**A**) Western blot analysis of wild-type (WT) and mutant RBD-Lg-His. (**B**) Mutations of RBD on its ACE2-binding ability. In vitro luciferase assay was carried out as described in Figure 1E. (**C**) Mutations of RBD on their response to antibody. Protein lysates extracted from WT or mutant RBD-Lg-His were preincubated with 50 µg/mL of IgG or VHH72, and subsequently incubated with Sm-ACE2 lysate, followed by luciferase assay. (**D**) Mutations of RBD on their response to peptide inhibition. Experimental procedures and data analysis are as described in Figure 3D. *, *p* < 0.05.

Monoclonal antibodies have previously been shown to be able to bind to SARS-CoV-2 RBD and disrupt RBD-ACE2 interaction, which inhibits viral entrance into cells [22]. We used a VHHR72 anti-RBD antibody, which has previously been shown to effectively inhibit RBD-ACE2 PPI [10,23], to test our biosensor. The biosensor activity was suppressed by VHH72 rather than IgG control in a dose-dependent manner (Figure 4B). We next synthesized peptides based on the RBD-ACE2 complex structure and peptide–protein docking ranking [8,14].

Although ACE2-P1 had no effect on the SRAE2-BS activity, ACE2-P2 significantly suppressed SRAE2-BS with an IC50 of 42 µM (Figure 3C). Interestingly, different from ACE2-derived peptides targeting virus RBD, the peptides RBD-P1, P2, P3 targeting ACE2 significantly suppressed SRAE2-BS activity in a dose-dependent manner (Figure 3D) with IC50 values of 9.0, 6.8, and 1.1 µM, respectively. Our findings clearly show that our SRAE2-BS can be a very useful and simple tool to quickly validate SMs, antibodies, and peptides disrupting RBD-ACE2 PPI for anti-COVID-19 therapy. One character of coronavirus is the high frequency of mutations during spreading.

Although the mutation rate for SARS-CoV-2 is not very high, many mutations have been detected in patients all over the world. However, as there is no system for the rapid analysis of these mutations, how these mutations affect their binding to ACE2 is largely unknown. By taking advantage of our newly constructed SRAE2-BS, we analyzed some of the RBD mutants detected in COVID-19 patients worldwide [24,25,26,27,28,29]. Except for the RBD-G476S mutant, which is not stable in cells, compared to RBD-WT, western blot analysis indicated that the expression levels of RBD mutants were similar (Figure 4A).

When the same amounts of Sm-ACE2-His protein lysates were mixed with those of WT or each mutant RBD, RBD-L452A and RBD-T572I are similar to RBD-WT in binding to ACE2, whereas the A474V, G476S, and F490L mutants in RBD significantly reduced its ability to bind to ACE2 (Figure 4B). Most interestingly, compared to WT RBD, the V367F, L452R, and Q493A RBD mutants obtained a three-to-five-fold increase in their binding to ACE2 (Figure 4B), suggesting that some of the RBD mutants indeed enhance the binding of the virus to its receptor, therefore, likely resulting in higher entrance into cells and infectibility.

Consistent with our findings, SARS-CoV-2 with V367F or L452R mutations was indeed shown to have enhanced entrance into cells, whereas the virus with G476S, A475V or F490L mutations demonstrated reduced entrance into cells [28]. Our findings provide strong evidence that our SRAE2-BS is a surrogate assay for evaluating the ability of SARS-CoV-2 RBD mutants on ACE2 binding and virus infectibility. We next tested whether the mutants with enhanced binding to ACE2 were also sensitive to antibody suppression. As shown in Figure 4C, although the binding of V367F and L453R mutants to ACE2 were still significantly suppressed by VHH72 at the same levels as WT RBD, whereas the Q493A mutant was relatively insensitive to antibody suppression (Figure 4C).

We also tested whether the most potent peptide disrupting RBD-ACE2 PPI, RBD-P3, was able to suppress mutant RBD activity (Figure 3D). Significantly, RBD-P3 can suppress the interaction of ACE2 with both WT and mutants at similar IC50 values (Figure 4D). As a control, the RBD-P3 peptide had little effect on the LATS kinase biosensor, Sm-YAP/LgTEAD (Figure 4D), suggesting that the effect of RBD-P3 on RBD:ACE2 PPI was specific. Together, these findings clearly suggest that our SRAE2-BS is a useful tool for the biochemical analysis of virus RBD mutants and their sensitivity to drug treatments.

## 4. Conclusions

In this study, we developed an ultrasensitive, simple, rapid biochemical biosensor tool to analyze the interactions of virus RBD and human ACE2 both in living cells and in vitro. This will have significant implications and applications in both basic research on SARS-CoV-2/ACE2 biology and COVID-19 clinical diagnosis and therapeutic drug discovery.

## Supplementary Materials

The following are available online at https://www.mdpi.com/article/10.3390/v13061055/s1. Table S1, Primers used for construction of RBD:ACE2 NanoBiT biosensor. Table S2, Primers used for deletions and mutagenesis.
