# Supplementary material for: A Bioluminescent Biosensor for Quantifying the Interaction of SARS-CoV-2 and Its Receptor ACE2 in Cells and In Vitro
Source: Viruses. 2021 Jun 2;13(6):1055. doi: 10.3390/v13061055 (PMC8227885; doi:10.3390/v13061055)
Supplement: Supplementary file 1 [file viruses-13-01055-s001.zip › viruses-1221580-supplementary.pdf]

An Ultrasensitive Biosensor for Quantifying the Interaction of SARS-CoV-2 and Its Receptor ACE2 in Cells and *in vitro*

Xiaolong Yang, Lidong Liu, Yawei Hao, Eva So, Sahar Sarmasti Emami, Derek Zhang, Yanping Gong, Prameet Sheth, Yu Tian Wang

This supporting information includes 2 tables for sequences of primers used for biosensor constructions and mutagenesis.

Table 1S. Primers used for construction of RBD:ACE2 NanoBiT biosensor

No	Name	Primer sequence
1	EcoRV-SmBiT-GS-F	5'-CCGGATATCAGTGACCGGCTACCGGCTGTTCGAGGAGATTCTCGGGAGTTCCGGTGGTGGCGGGAGCGGAGGTGGAGGCTCGAG CGGT-3'
2	GS-ACE2-18F	GGGAGTTCCGGTGGTGGCGGGAGCGGAGGTGGAGGCTCGAGCGGTCAGTCCACCATTGAGGAACAG
3	NcoI-STOP-6HisG-ACE2-615R	5'-CATGCCATGGTTAATGGTGATGGTGATGATGTCCGTCTGCATATGGACTCCAGT-3'
4	EcoRV-ACE2-18F	5'-CCGGATATCACAGTCCACCATTGAGGAACAG-3'
5	GS-ACE2-615R	5'-ACCGCTCGAGCCTCCACCTCCGCTCCCGCCACCACCGGAACTCCCGTCTGCATATGGACTCCAGT-3'
6	NcoI-STOP-6HisG-SmBiT-GS-R	5'-CATGCCATGGTTAATGGTGATGGTGATGATGTCCGAGAATCTCCTCGAACAGCCGGTAGCCGGTCACACCGCTCGAGCCTCCACCTCCGCTCCCGCCACCACCGGAACTCCC-3'
7	EcoRV-LgBiT-F	5'-CCGG ATA TCA GTCTTCACACTCGAAGATTTC-3'
8	GS-LgBiT-R	5'-ACCGCTCGAGCCTCCACCTCCGCTCCCGCCACCACCGGAACTCCCACTGTTGATGGTTACTCGGAACAG-3'
9	GS-RBD319-F	5'- GGGAGTTCCGGTGGTGGCGGGAGCGGAGGTGGAGGCTCGAGCGGTAGGGTCCAACCAACAGAGAGC-3'
10	NcoI-STOP-6HisG-RBD-541R	5'-CATGCCATGGTTAATGGTGATGGTGATGATGTCCGAAGTTCACACACTTGTTCTTC-3'
11	EcoRV-RBD319-F	5'- CCGG ATA TCA AGGGTCCAACCAACAGAGAGC-3'
12	GS-RBD-685R	5'- ACCGCTCGAGCCTCCACCTCCGCTCCCGCCACCACCGGAACTCCCCCTTGCCCTCCTTGGGCTG-3'
13	NcoI-STOP-6HisG-LgBiT-R	5'-CATGCCATGGTTAATGGTGATGGTGATGATGTCCACTGTTGATGGTTACTCGGAACAG-3'
14	NcoI-STOP-6HisG-ACE2-515R	5'-CATGCCATGGTTAATGGTGATGGTGATGATGTCCATATCGAATGAATGAGTAATC-3'
15	NcoI-STOP-6HisG-RBD-685R-NoStop	CATGCCATGGTTAATGGTGATGGTGATGATGTCCCCTTGCCCTCCTTGGGCTGTTG
16	GS-LgBiT-F	5'-GGCTCGAGCGGTGGTGGCGGGAGCGGAGGTGGAGGGTCGTCAGGTGTCTTCACACTCGAAGATTTC

Table S2. Primers used for deletions and mutagenesis

No	Name	Primer Sequence
1	NcoI-STOP-6HisG-RBD-541R	5'- CATGCCATGGTTAATGGTGATGGTGATGATGTCCGAAGTTCACACACTTGTTCTTC-3'
2	GS-RBD541-R	5'- ACCGCTCGAGCCTCCACCTCCGCTCCCGCCACCACC GGA ACTCCCGAAGTTCACACACTTGTTCTTC-3'
3	NcoI-STOP-6HisG-ACE2-515R	5'- CATGCCATGGTTAATGGTGATGGTGATGATGTCCATATCGAATGAATGAGTAATC-3'
4	NcoI-STOP-6HisG-ACE2-415R	5'-CATGCCATGGTTAATGGTGATGGTGATGATGTCCAGGTGTGGCTGCAGAAAGTGAC-3'
5	ACE2-K31A-S	5'- CAAGACATTTTTGGACGCGTTTAACCACGAAGCC-3'
6	ACE2-K31A-AS	5'- GGCTTCGTGGTTAAACGCGTCCAAAAATGTCTTG-3'
7	ACE2-E35A-S	5'-GACAAGTTTAACCACGCAGCCGAAGACCTGTTC-3'
8	ACE2-E35A-AS	5'- GAACAGGTCTTCGGCTGCGTGGTTAAACTTGTC-3'
9	ACE2-M82A-S	5'-GTCCACACTTGCCCAAGCGTATCCACTACAAGAAATTC-3'
10	ACE2-M82A-AS	5'-GAATTTCTTGTAGTGGATACGCTTGGGCAAGTGTGGAC-3'
11	ACE2-K353A-S	5'-GCTTGGGACCTGGGGGCGGGCGACTTCAGGATCC-3'
12	ACE2-K353A-AS	5'-GGATCCTGAAGTCGCCCGCCCCCAGGTCCCAAGC-3'
13	RBD-V367F-S	5'-GTGTGGCTGACTACTCTTTCCTCTACAACTCTGCCTC-3'
14	RBD-V367F-AS	5'- GAGGCAGAGTTGTAGAGGAAAGAGTAGTCAGCCACAC-3'
15	RBD-L452R-S	5'- GGCAACTACAACTAC*CGC*TACAGACTGTTCAG-3'
16	RBD-L452R-AS	5'- CTGAACAGTCTGTA*GCG*GTAGTTGTAGTTGCC-3'
17	RBD-L455A-S	5'-CAACTACCTCTACAGAGCGTTCAGGAAGAGCAAC-3'
18	RBD-L455A-AS	5'-GTTGCTCTTCCTGAACGCTCTGTAGAGGTAGTTG-3'
19	RBD-A475V-S	5'- CAGAGATTTACCAG*GTT*GGCAGCACACCATG-3'
20	RBD-A475V-AS	5'-CATGGTGTGCTGCCAACCTGGTAAATCTCTG-3'

21	RBD-G476S-S	5'-GAGATTTACCAGGCTAGCAGCACACCAT*GT*AATG-3'
22	RBD-G476S-AS	5'-CATTACATGGTGTGCTGCTAGCCTGGTAAATCTC-3'
23	RBD-F490L-S	5'-GGGCTTCAACTGTTACCTTCCACTCCAATCCTAT-3'
24	RBD-F490L-AS	5'-ATAGGATTGGAGTGGAAGGTAACAGTTGAAGCCC-3'
25	RBD-Q493A-S	5'-CTGTTACTTTCCACTCGCATCCTATGGCTTCCAACC-3'
26	RBD-Q493A-AS	5'-GGTTGGAAGCCATAGGATGCGAGTGGAAAGTAACAG-3'
27	RBD-T572I-S	5'-GGGACATTGCTGACATCACAGATGCTGTGAG-3'
28	RBD-T572I-AS	5'-CTCACAGCATCTGTGATGTCAGCAATGTCCC-3'
